# Longitudinal associations between family conflict, intergenerational transmission, and adolescents’ depressive symptoms: evidence from China Family Panel studies (2016–2020)

**DOI:** 10.1186/s13034-025-00866-9

**Published:** 2025-02-17

**Authors:** Yu Jin, Jiayi Liu, Pan Li, Yunquan Hu, Xintian Hong, Xiaoliang Li, Yongyong Teng, Mingxing Huang, Yuanyuan Wang

**Affiliations:** 1 Department of Statistics, Faculty of Arts and Sciences, Beijing Normal University, Beijing, China; 2 Department of Mathematics, Beijing Normal University, Beijing, China; 3 Key Laboratory of Brain, Cognition and Education Sciences, School of Psychology, Center for Studies of Psychological Application, and Guangdong Key Laboratory of Mental Health and Cognitive Science, Ministry of Education, China, South China Normal University, Guangzhou, China; 4 The Third People’s Hospital of Zhuhai, Zhuhai, China

**Keywords:** Depression, Family conflict, Intergenerational transmission, Longitudinal study, CFPS

## Abstract

**Background:**

Adolescent depression is increasing globally, and family conflict may contribute to its transmission across generations. However, longitudinal evidence on these dynamics remains sparse. This study examines the longitudinal associations between family conflict and adolescents’ and parents’ depressive symptoms from three waves of data.

**Methods:**

Data from the 2016–2020 China Family Panel Studies (CFPS) were analyzed, including 1,772 adolescents (Mean age = 12.4, SD = 1.68 in 2016) and their parents. Family conflict was measured using three questions from adolescents, while depressive symptoms were assessed using the Center for Epidemiological Studies Depression Scale (CESD). Multiplelinear regression, latent growth models (LGM), and cross-lagged panel models (CLPM) were employed to examine longitudinal associations between family conflict and depressive symptoms.

**Results:**

The results revealed that family conflict correlated with adolescents’ depressive symptoms (*r = 0.580*,*p < 0.001*). Adolescents’ depressive symptoms also exacerbated family conflict (*β1* = 0.030, *p* < 0.05; *β2* = 0.032, *p* < 0.01) across three waves, while family conflict had a limited contribution to parents’ depressive symptoms. Mothers’ depressive symptoms influenced adolescents’ depressive symptoms significantly (*β = 0.043*,*p < 0.05*), while adolescents’ depressive symptoms were transmitted to fathers’ depressive symptoms (*β = 0.080*,*p < 0.01*) between Wave 2 and Wave 3. Moreover, the mother’s education level negatively predicted adolescents’ depressive symptoms (*β = -0.296*,*p < 0.05*).

**Conclusions:**

Family conflict plays a critical role in adolescents’ depressive symptoms and its intergenerational transmission. The findings underscore the pivotal role of family dynamics in mental health, especially in the development of adolescents’ depressive symptoms. Interventions aimed at reducing family conflict may help mitigate depressive symptoms across generations.

**Supplementary Information:**

The online version contains supplementary material available at 10.1186/s13034-025-00866-9.

## Introduction

Adolescent depression is a global public health concern with increased prevalence, which increased from 24% during the period from 2001 to 2010 to 37% between 2011 and 2020 globally [1, 2]. In China, the prevalence of depressive symptoms among adolescents increased during the decades, ranging from 24.30% to 28.23% [3, 4]. Moreover, adolescent depression is associated with a range of negative consequences, including chronic diseases, mental disorders, and poor social relationships and prospects [5]. It often results in extensive psychosocial and vocational impairments with damaging long-term effects [6]. Therefore, it is crucial to develop and implement effective prevention strategies and early interventions for this vulnerable group.

Previous studies have found that several factors are associated with adolescent depression, including family conflict and parental depression [7, 8]. Family conflict (e.g., parent-child, interparental) has garnered escalating interest as a salient factor in early-onset depression [7, 9]. Adolescents in high-conflict households are more prone to depressive symptoms like withdrawal and emotional dysregulation [10–12], which may persist into adulthood [13]. Such conflict also weakens parent-child bonds, reducing communication quality and emotional support [14], leading to feelings of isolation and worthlessness in adolescents. Additionally, adolescents may internalize aggressive or avoidant behaviors observed in conflict-ridden homes, adopting maladaptive coping strategies that contribute to depression [15, 16]. Reciprocally, adolescent depression can elicit negative parenting styles, intensifying family conflict and perpetuating a cycle of family-related depression [17].

Furthermore, there exists intergenerational transmission of parents’ depression and adolescents’ depression besides psychopathological genetic influences [18, 19]. For example, a systematic review and meta-analysis found that the prevalence of depression among subsequent generations of adolescents is 2.5 times that of their parents at the age of 15. They are 4.21 times more likely to meet the criteria for major depressive disorder than adolescents whose parents have never been depressed [20]. This intergenerational transmission is influenced by hereditary pathways and bidirectional interactions, where both parents’ and adolescents’ depression reciprocally shape one another [21, 22]. Contextual variables such as socioeconomic status [23], marital stability [24], and cultural or racial backgrounds [25] further exacerbate these dynamics, underscoring the multifaceted and disruptive nature of family depression.

Moreover, Chinese culture also influences the association between family conflict, parents’ depression, and adolescents’ depression through various cultural concepts and values. In Chinese familial contexts, parent-adolescent relationships are typically hierarchical and authority-driven, with parents exerting substantial influence over their offspring’s lives [26]. The deeply rooted concept of filial piety, mandating adolescent respect and obedience to parents [27, 28], often constrains open communication and emotional disclosure, hindering adolescents from articulating their feelings or concerns. This communication deficit, when compounded by family conflict or parental depression, can amplify adolescents’ sense of isolation and depression [22]. Additionally, the Chinese cultural emphasis on collectivism and family centrality positions the family as the paramount source of support and guidance, with individual welfare closely linked to the family collective. Consequently, family conflict can exert a profound impact on all members, including adolescents [29], disrupting family harmony and inducing heightened stress and emotional turmoil, which may precipitate depression in both parents and adolescents [30].

While the extensive research on the association between family conflict, parents’ depression, and adolescents’ depression has generally yielded consistent findings, the underlying mechanisms and causative pathways remain unclear. Prior cross-sectional studies have identified bidirectional relationships: family conflict heightens the risk of adolescents’ depression, and conversely, adolescents’ depression can also lead to family conflict [17, 31]. Moreover, from a lifelong perspective, adolescents’ depression can be viewed as a dynamic condition influenced by time-varying factors [32, 33], highlighting the importance of its developmental aspect in understanding its progression and complexity. However, cross-sectional studies offer limited insights into adolescents’ depression, making it challenging to establish causality or elucidate the longitudinal associations among family conflict, parents’ depression, and adolescents’ depression [34]. Longitudinal studies address these limitations by tracking changes over time and examining the predictive association between family conflict and the intergenerational transmission of depression. They also enable the identification of individual trajectories rather than focusing solely on group differences at a single time point [35]. Furthermore, appropriate methods for longitudinal data can account for interindividual heterogeneity to yield more consistent estimates. When investigating the longitudinal link between family conflict and adolescents’ depression, it is crucial to adjust for potential confounders such as age, gender, parental education levels, and parents’ marital levels.

Therefore, we intend to conduct longitudinal analyses leveraging three waves of data from the Chinese Family Panel Study (CFPS) to examine the association between family conflict and adolescents’ depressive symptoms and the intergenerational transmission of these symptoms. The CFPS, a nationally representative, comprehensive, longitudinal survey in China, employs a multi-stage probability sampling method to ensure sample representativeness. In this study, we utilized latent growth models (LGM) and cross-lagged panel models (CLPM) to (1) explore the trajectories of conflict and depressive symptoms within families over time, (2) assess the contagion effects of depressive symptoms among family members, and (3) uncover the mechanisms of underlying intergenerational transmission and its amplification through family conflict. This approach will advance our understanding of how family conflict contributes to the dynamics of family depressive symptoms, providing valuable insights for designing targeted interventions to break the cycles of intergenerational depressive symptoms and improve family well-being.

## Methods

### Study design, participants, and data collection

**Fig. 1:**
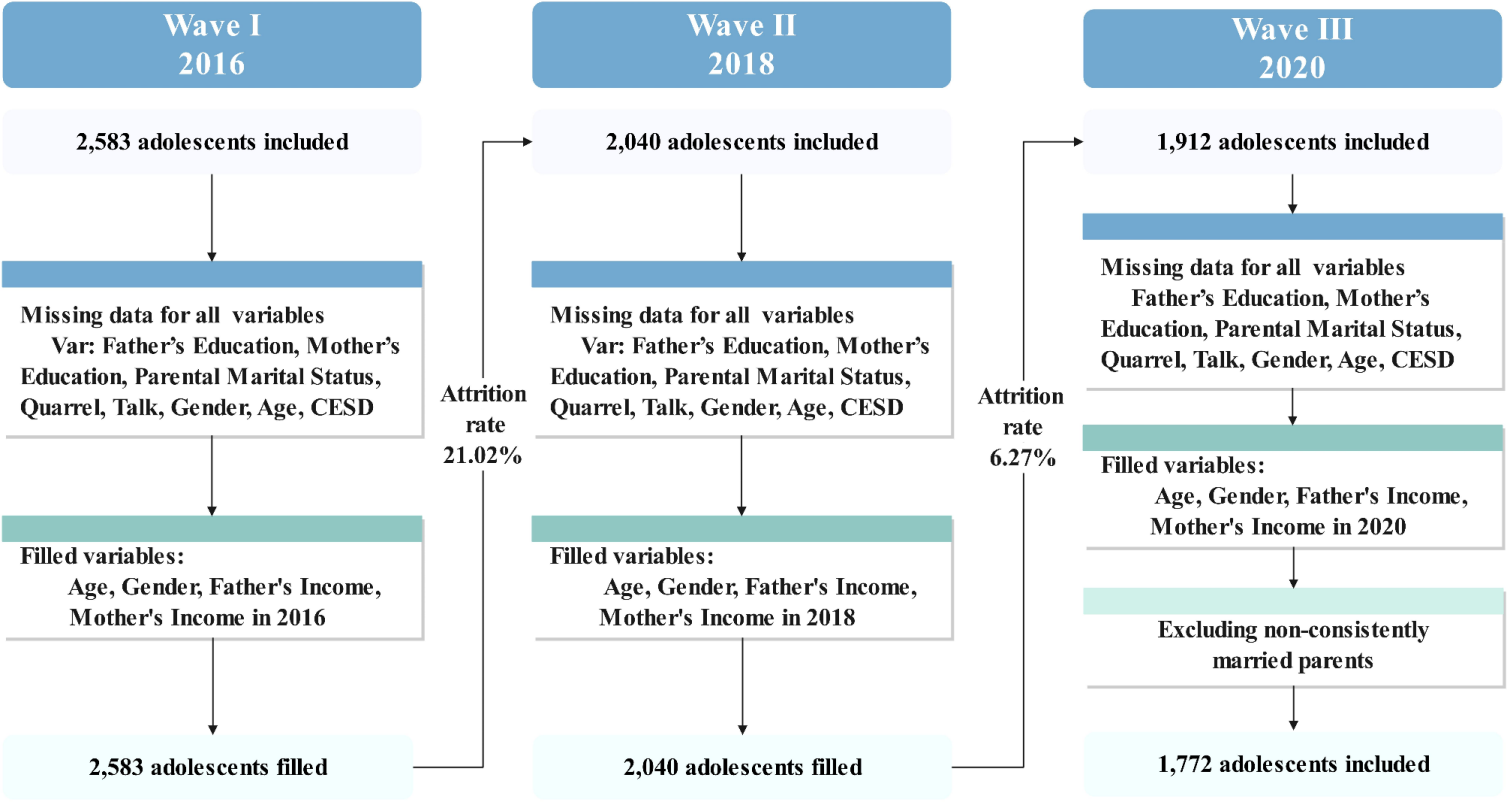
Flow chart of sample selection

This study utilized data from the China Family Panel Study (CFPS), a nationally representative, comprehensive, longitudinal survey conducted by the Institute for Social Science Surveys (ISSS) at Peking University, China (https://www.isss.pku.edu.cn/cfps/). The 2010 CFPS baseline survey sample was selected through a multi-stage probability approach with implicit stratification, designed to reduce operational costs and facilitate social context studies. The sampling process involved three stages: county (or equivalent), village (or equivalent), and household levels. The target population includes all family members in households in these 25 provinces. A “household” in the CFPS refers to an economically independent dwelling unit with at least one Chinese national family member (excluding Hong Kong, Macao, and Taiwan). All members over age 9 in a sampled household are interviewed and constitute the core members of the CFPS. During the 2010 baseline survey, nearly 15,000 households and nearly 30,000 individuals were interviewed, with an approximate response rate of 79%. Follow-up surveys have been conducted biennially since 2012. For this study, data from three CFPS waves (2016, 2018, and 2020) were analyzed, encompassing the Family Member Relationship, Family Economics, and Individual Self-Response databases. Personal identifier (PID) codes matched participants and households across waves. In the CFPS adolescents’ database, the age range is 9 to 15 years old, but only those aged 10–15 completed self-response questionnaires, including the CESD and family conflict scales. Thus, adolescents not aged 10–15 in 2016, participants with substantial missing data, and families participating only in the first wave were excluded. The sample size decreased from 2,583 adolescents in 2016 to 2,040 in 2018 and 1,912 in 2020. Additionally, only samples where parents maintained a consistently married status from 2016 to 2020 were retained. Consequently, the final sample included 1,772 adolescents (Mean age = 12.4, SD = 1.68 in 2016) and their parents, who participated in all three survey waves. Figure 1 illustrates the sample selection process.

### Variables

#### Depressive symptoms

Depressive symptoms were measured using the Chinese version of the Center for Epidemiologic Studies Depression Scale (CESD) [36, 37], which is widely used in epidemiological and clinical research. In the initial CFPS datasets, each item of CESD was coded from “1” (rarely) to “4” (most of the time). In order to follow the original CESD scale, we recoded each item from “0” (rarely) to “3” (most of the time). The higher total scores of CESD indicate more severe depressive symptoms. For each family, the depressive symptoms of every participant were assessed separately from fathers, mothers, and adolescents during each round. The Cronbach’s alpha coefficients of the CESD-8 for the 2016, 2018, and 2020 rounds were 0.93, 0.95, and 0.96, respectively.

#### Family conflict

Family conflict was assessed using three questions from adolescents: (1) “How often did you quarrel with your parents in the past month?” (2) “How often did your parents quarrel in the past month?” and (3) “How often did you have heart-to-heart talks with your parents in the past month?” Quarrels with parents were rated on a 0- to 2-point scale (0 = no quarrel, 1 = 1–2 quarrels, 2 = more than three quarrels). Parents’ quarrels were rated on a 0- to 2-point scale (0 = no quarrel, 1 = 1–2 quarrels, 2 = more than three quarrels). Heart-to-heart talks with parents were reverse-scored (0 = more than four talks, 1 = 2–3 talks, 2 = 0–1 talk). The total score ranged from 0 to 6, with higher scores indicating more conflict. Scores were categorized as no conflict (0), medium conflict (1–3), or high conflict (4–6).

#### Control variables

To account for potential confounding factors between family conflict and depressive symptoms, we included several control variables: adolescent’s age, gender (male or female), and parents’ education levels (elementary or lower, middle or high school, university or higher). We also included parents’ current and total annual family income, measured by the question, “What is your household’s current annual income?” These variables were all self-reported.

### Data analytic plan

Predictive mean matching (PMM) method was employed to address missing data. Correlation analysis was employed to investigate the associations between family conflict and depressive symptoms. Latent growth models (LGM) and cross-lagged panel models (CLPM) were used to explore the longitudinal relationships and interactions between family conflict and depressive symptoms. All data analyses were performed using R version 4.3.3.

#### Imputation of missing data

Missing data were addressed using multiple imputations with the PMM method [38], especially in datasets with a mix of continuous and categorical variables. This approach treated the missing data as occurring randomly and used variables such as age, gender, and parents’ income to estimate missing values. We also applied the K-nearest neighbors (KNN) and random forest methods for imputing missing data to assess the influence of imputation on the study results.

#### Correlation and multiple Linear regression

Spearman’s correlation and multiple linear regression were used to investigate relationships between family conflict and depressive symptoms. Three regression models were developed to assess the impact of family conflict on the depressive symptoms of adolescents, mothers, and fathers across 2016, 2018, and 2020. Family conflict was the primary independent variable, with CESD scores as the dependent variable. Covariates included adolescent’s age, adolescent’s gender and parents’ education levels.

#### Latent growth models

LGM were used to assess the trajectory of depressive symptoms and family conflict over time. This method, a subset of Structural Equation Models, captures individual variations in growth patterns through random effects. The model included an intercept (initial level) and a slope (rate of change) over the three survey waves. Fixed loadings were set at 0, 1, and 2, corresponding to the two-year intervals. Separate linear growth models were developed for adolescents, mothers, and fathers, adjusting for parents’ education levels and marital statuses.

#### Cross-lagged panel models

CLPM were used to examine the reciprocal relationship between family conflict and depressive symptoms over time. CLPM allows for analyzing directional influences between variables at different time points, providing insight into potential causal relationships. Age and gender were considered as covariates in this model.

## Results

### Descriptive statistics of participants

Table 1 presents the demographic characteristics of adolescents and their parents. CESD scores of adolescents were 3.8 (*SD = 2.83*) in 2016, 4.2 (*SD = 3.10*) in 2018, and 4.7 (*SD = 3.70*) in 2020. Significant differences were found among these variables, including CESD scores of adolescents and their parents, family conflict, parents’ income, adolescent-parent quarrels, parental quarrels, and heart-to-heart talks between adolescents and parents over the three years (all *p* < 0.001).

### The result of correlation and multiple linear regression

**Fig. 2:**
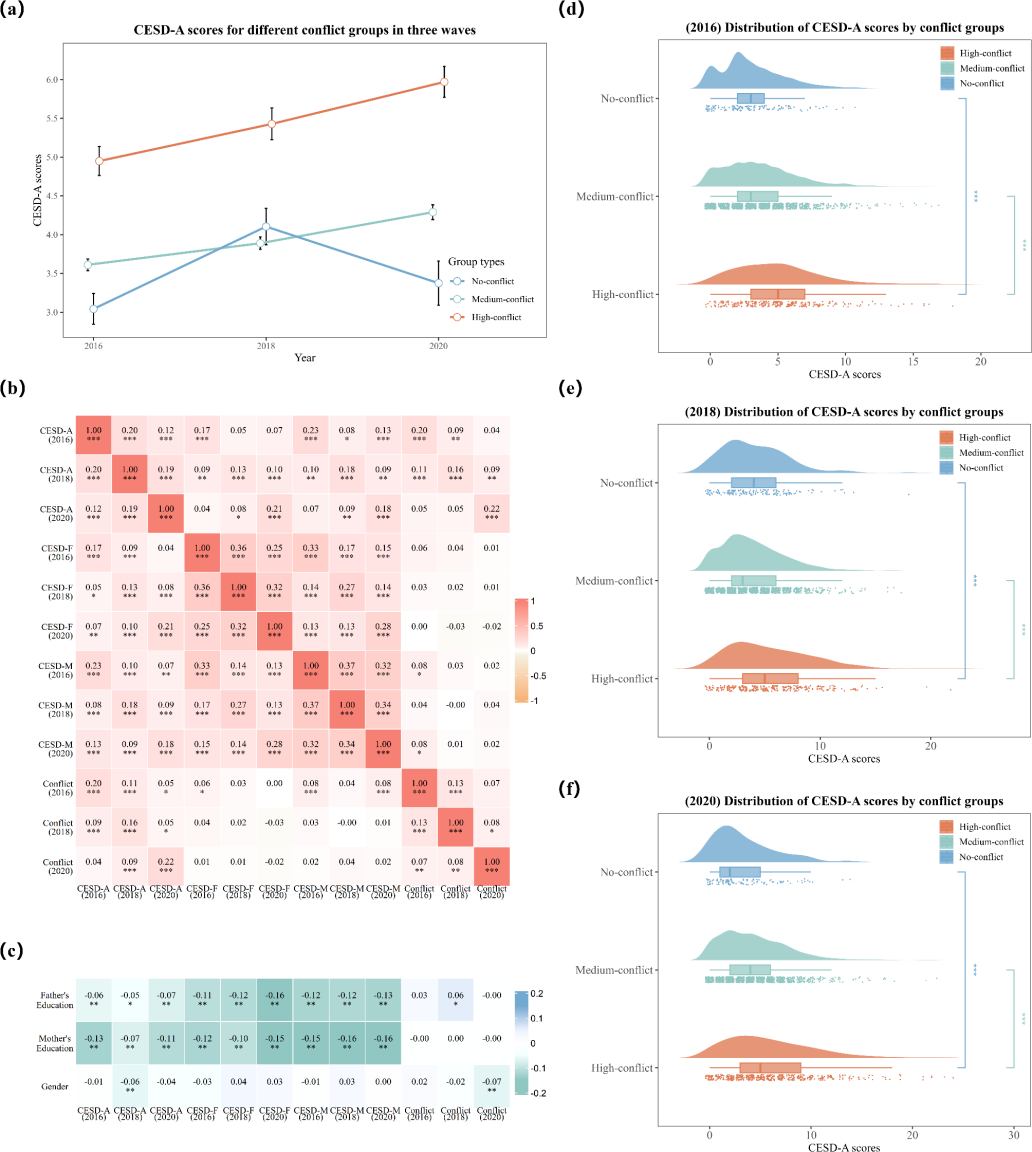
(a) CESD-A scores for different conflict groups in three waves; (b) Correlation matrix for CESD scores of adolescents, parents and family conflict in three waves; (c) Correlation matrix for CESD scores of adolescents and parents, family conflict and control variables; (d) Raincloud plot of CESD-A scores for three conflict groups in 2016; (e) Raincloud plot of CESD-A scores for three conflict groups in 2018; (f) Raincloud plot of CESD-A scores for three conflict groups in 2020

*Notes*. CESD-A, CESD scores of adolescents; CESD-F, CESD scores of fathers; CESD-M, CESD scores of mothers.

The correlation matrices demonstrated positive correlations in depressive symptoms among family members across 2016, 2018, and 2020. Both mother’s and father’s education levels showed negative correlations with depressive symptoms across all family members during the three time points (Fig. 2b and c). Multiple linear regression revealed family conflict was a significant predictor of adolescents’ depressive symptoms (*β = 0.46*, *p < 0.001*,* Adjusted R*^*2*^ *= 0.054*), and mothers (*β = 0.10*,* p < 0.05*,* Adjusted R*^*2*^ *= 0.002*), indicating that family conflict had a stronger impact on adolescents’ mental health. Figure 2d, e and f illustrate the distributions of CESD scores of adolescents across three conflict groups (no conflict, medium conflict, and high conflict) over three years.

**Table 1:** Demographic characteristics of adolescents and their parents in three waves (2016, 2018, 2020)

	Wave 2016	Wave 2018	Wave 2020	Chi-square/F Statistics	*P* value	Partial η^2^
	(*N* = 1,772)	(*N* = 1,772)	(*N* = 1,772)			
	*n* (%)					
**Sex**				-	-	-
Female	819 (46.2%)	819 (46.2%)	819 (46.2%)			
Male	953 (53.8%)	953 (53.8%)	953 (53.8%)			
**Father**’**s education level**				-	-	-
Elementary school or below	798 (45.0%)	798 (45.0%)	798 (45.0%)			
Middle to high school	927 (52.3%)	927 (52.3%)	927 (52.3%)			
University or above	47 (2.7%)	47 (2.7%)	47 (2.7%)			
**Mother**’**s education level**				-	-	-
Elementary school or below	1008 (56.9%)	1008 (56.9%)	1008 (56.9%)			
Middle to high school	726 (41.0%)	726 (41.0%)	726 (41.0%)			
University or above	38 (2.1%)	38 (2.1%)	38 (2.1%)			
	***mean (SD)***					
**Age**	12.4 (1.68)	14.4 (1.68)	16.4 (1.68)	-	-	-
**Father**’**s income (yuan)**	10,611 (29,731)	29,908 (16,555)	33,450 (23,147)	13.71	< 0.001	0.31
**Mother**’**s income (yuan)**	2248 (4511)	17,130 (12,188)	13,487 (11,718)	26.51	< 0.001	0.72
**CESD-A**	3.8 (2.83)	4.2 (3.10)	4.7 (3.70)	38.28	< 0.001	0.04
**CESD-F**	4.6 (3.60)	5.3 (3.73)	5.4 (4.01)	35.87	< 0.001	0.04
**CESD-M**	5.4 (3.82)	6.1 (4.00)	6.1 (3.97)	26.14	< 0.001	0.03
**Family conflict**	2.30 (1.30)	2.33 (1.36)	2.65 (1.44)	35.01	< 0.001	0.04
Adolescent -parent quarrels	0.50 (0.74)	0.53 (0.74)	0.73 (0.81)	47.71	< 0.001	0.05
Parental quarrels	0.80 (2.07)	0.83 (2.24)	1.38 (3.58)	18.90	< 0.001	0.02
Heart-to-heart talks	1.42 (0.78)	1.41 (0.79)	1.35 (0.76)	5.34	< 0.001	0.01

*Notes.* SD, standard deviation; CESD-A, CESD scores of adolescents; CESD-F, CESD scores of fathers; CESD-M, CESD scores of mothers; Partial η^2^, the effect size of repeated measures one-way ANOVA

### The result of latent growth models

**Fig. 3:**
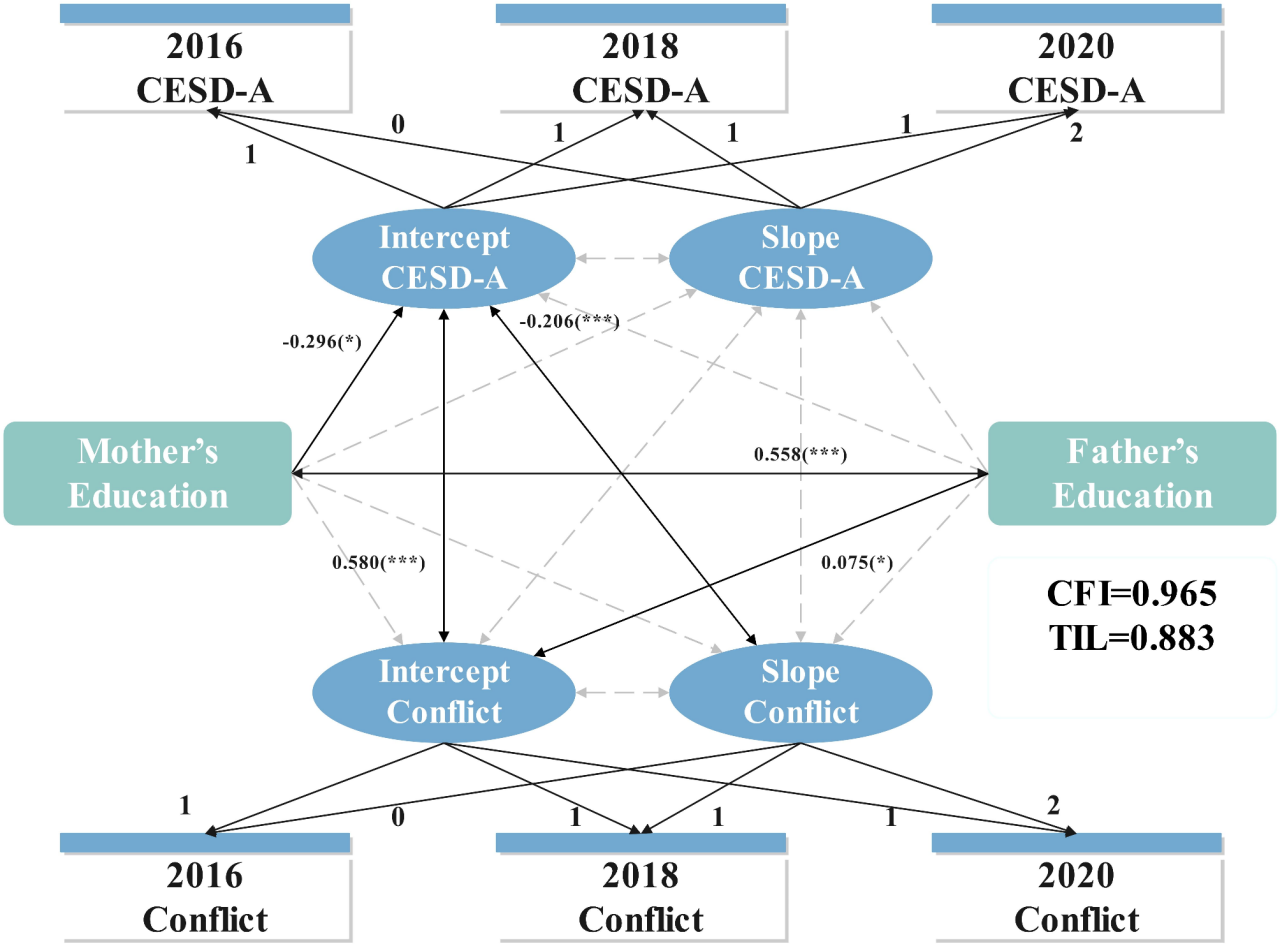
Latent growth model of adolescents’ depressive symptoms and family conflict

*Notes.* CESD-A, CESD scores of adolescents; CFI, comparative fit index; TIL, tucker-lewis index.

The LGMs for adolescents showed good fit indices, with significant variances in intercepts and slopes (*CFI = 0.965*,* TLI = 0.883*,* RMSEA = 0.033*). Figure 3 displays that adolescents’ depressive symptoms intercept had a strong positive association with family conflict intercept (*r = 0.580*, *p < 0.001*), demonstrating the cumulative impact of family conflict on adolescents’ depressive symptoms. Furthermore, the mother’s education level was negatively associated with adolescents’ depressive symptoms (*β = -0.296*, *p < 0.05*). In addition, the father’s education level was positively associated with family conflict (*β = 0.075*, *p < 0.05*).

The growth trajectories and associated pathways among parents’ models (mothers and fathers; supplemental materials Figures A1 and A2) demonstrated patterns similar to those observed in the adolescent model.

### The result of cross-lagged panel models

**Fig. 4:**
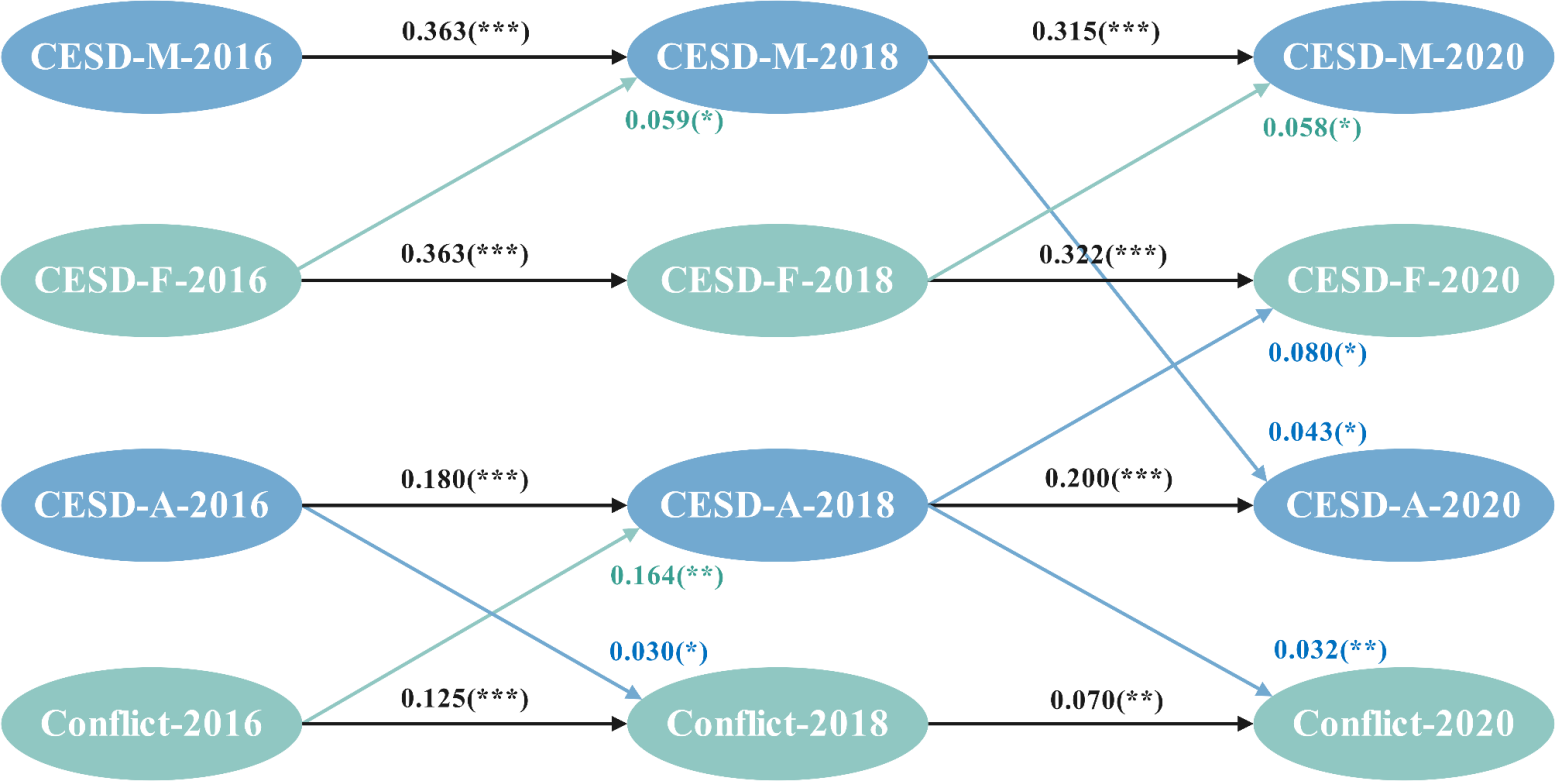
Cross-lagged panel model for depressive symptoms and family conflict

*Notes*. CESD-A, CESD scores of adolescents; CESD-F, CESD scores of fathers; CESD-M, CESD scores of mothers.

The final CLPM fitted the data well (*CFI = 0.808*,* SRMR = 0.041*,* RMSEA = 0.080 [0.073*,* 0.087]*). Figure 4 highlights significant transmission effects of depressive symptoms and family conflict among family members across time. The model identified substantial autoregressive effects for adolescents’, fathers’, and mothers’ depressive symptoms and family conflict, which means these variables were significantly associated with their values of last year (*p < 0.001*). Family conflict significantly affected adolescents’ depressive symptoms (*β* = 0.164, *p* < 0.01) between Wave1 and Wave2. Adolescents’ depressive symptoms also exacerbated family conflict (*β1* = 0.030, *p* < 0.05; *β2* = 0.032, *p* < 0.01) across three waves, suggesting a significant and stable bidirectional relationship between family conflict and adolescents’ depressive symptoms, whereas family conflict appeared to have a limited contribution to parents’ depressive symptoms. Furthermore, key pathways included depressive symptoms transmission from “father to mother”, “mother to adolescent”, and “adolescent to father” at different time points. Specifically, mothers’ depressive symptoms influenced adolescents’ depressive symptoms significantly (*β = 0.043*,*p < 0.05*), while adolescents’ depressive symptoms were transmitted to fathers’ depressive symptoms (*β = 0.080*, *p < 0.01*) between Wave 2 and Wave 3. The results showed that depressive symptoms exerted bidirectional influences on both parental interactions and parent-adolescent dynamics, indicating the complexity of their transmission within families.

### Sensitivity analysis

To ensure the robustness of the findings, we conducted sensitivity analyses to assess the influence of imputation on the study results. The PMM imputation method was applied for missing data handling. We performed multiple imputation runs with 5 different seeds to assess the variability of imputed values, and the results were consistent with this study. In addition, we also applied the K-nearest neighbors (KNN) and random forest methods for the imputation of missing data, which showed similar results to this study (supplemental materials Table S1, Table S2). The imputation methods did not result in significant differences in the outcomes, and more details were presented in supplemental materials Appendix A1. The consistency of results across various imputation methods bolsters the credibility of the findings, thereby supporting that the discerned effects are indicative of genuine underlying relationships rather than artifacts of the data handling techniques.

## Discussion

This study explored the longitudinal associations between family conflict and intergenerational depressive symptoms, utilizing a nationally representative sample from the China Family Panel Studies (CFPS) survey. The study examined how family conflict influences the development of depressive symptoms among adolescents and the subsequent intergenerational transmission of depressive symptoms within families. Based on latent growth models and cross-lagged panel models, results showed that family conflict correlated with adolescents’ depressive symptoms significantly, and mothers’ depressive symptoms influenced adolescents’ depressive symptoms.

Our research identified a significant longitudinal association between family conflict and depressive symptoms among adolescents, consistent with prior research [39–41]. Adolescents are in a critical stage of development where their sense of self and identity is being formed. During this period, they are susceptible to their environment and the relationships around them. When family conflict arises, it disrupts the stability and predictability of their home life, which is a crucial source of emotional security [42, 43]. Furthermore, family conflict often involves communication breakdowns and emotional unavailability, which can lead to feelings of isolation and loneliness in adolescents. These feelings can exacerbate depressive symptoms as the adolescent may lack the necessary emotional support to cope with their internal struggles [21, 22]. Moreover, the emotional intensity of family conflict can lead to chronic stress in adolescents. Chronic stress has been linked to the dysregulation of the hypothalamic-pituitary-adrenal (HPA) axis, which can result in the overproduction of stress hormones such as cortisol. Prolonged exposure to high cortisol levels can negatively affect brain development, particularly in areas related to emotional regulation and stress response, such as the prefrontal cortex and the hippocampus [44, 45]. These findings highlight the vulnerability of adolescents living in households with high levels of conflict and emphasize the need for interventions to strengthen parent-adolescent relationships to improve adolescents’ mental health [29]. Furthermore, Chinese culture significantly influences the association between family conflict and adolescents’ depressive symptoms. The concept of filial piety, emphasizing obedience to parental authority, expects adolescents to follow parental guidance without question, thereby limiting open communication and emotional expression [46]. For instance, a three-year longitudinal study revealed that authoritarian filial piety hinders cognitive autonomy development, positively contributing to adolescent depression [47]. This may increase the risk of depressive symptoms when adolescents quarrel with parents. Additionally, the hierarchical nature of parent-adolescent relationships in Chinese families can exacerbate conflict effects. Adolescents may feel powerless to express needs or emotions, leading to increased depression [48]. A longitudinal survey also found that family conflict and poor parent-adolescent relationships predict adolescents’ depressive symptoms [49]. The significant association between family conflict and adolescent depressive symptoms can be attributed to disrupted emotional security, chronic stress’s physiological effects, lack of emotional support, development of maladaptive coping mechanisms, and modeling of unhealthy conflict resolution strategies. Interventions such as family counseling, communication training, parenting skills training, and school-based programs can effectively mitigate the impact of family conflict on adolescent mental health.

The study also uncovered the intergenerational transmission of depressive symptoms, where symptoms in one family member influence others over time. The family atmosphere and emotional differentiation among members significantly impact mental health outcomes. Family environments marked by chronic stress, conflict, and a poor emotional climate can foster depression development [50]. Moreover, parental depression profoundly affects adolescents. Those with depressed parents may face less positive parenting, more conflict, and heightened family adversity, increasing their depression risk [51]. Shared family stressors, such as financial woes, health issues, or relationship problems, can also contribute to depressive symptoms across members. Adolescents, particularly vulnerable to their mother’s emotional state, align with research linking maternal distress to adolescent depression [52–54]. Maternal depression can shape adolescent depression trajectories, with youth stress responses significantly influencing risk and resilience. Additionally, maternal emotion regulation abilities affect adolescent depressive symptoms by mediating their own emotion regulation. A longitudinal study using network analysis found that maternal hostility most strongly influences adolescent mental health problems, while adolescent depression is most affected by maternal parenting practices. In Chinese families, the mother-child relationship is especially close, with mothers’ moods directly impacting adolescents’ mental health [55, 56]. Furthermore, adolescents with strong filial piety, a cultural value emphasizing parental respect, tend to experience higher depression levels [57, 58].

The mother’s education level is negatively associated with adolescent depressive symptoms, highlighting the role of broader social factors in mental health outcomes. Consistent with prior research, higher maternal education correlates with enhanced self-compassion in adolescents [59], improving their capacity to regulate emotions such as stress and anxiety [60]. A meta-analysis confirmed a significant negative association between maternal education and adolescent depressive symptoms [61]. Higher education can enhance parenting skills, including better communication and emotional support, and provide a stimulating learning environment for adolescents. Educated mothers may be more adept at providing emotional support and understanding, which can buffer against depressive symptoms in adolescents [62]. In addition, the father’s education level is positively associated with family conflict, underscoring the role of paternal educational attainment in family dynamics and mental health outcomes [63–65]. While higher education is generally associated with positive outcomes, some studies suggest that higher paternal education levels can be positively associated with family conflict. In Chinese culture, fathers with higher education levels may set higher academic and behavioral standards for their children, leading to increased pressure and conflict [64]. Additionally, demanding work schedules can diminish the quality and quantity of family interaction, fostering resentment and conflict [65]. Comprehending these mechanisms within their cultural and societal frameworks can guide policy and public health initiatives to enhance family dynamics and mental health outcomes.

These findings highlight the imperative for long-term, multifaceted interventions to address family conflict and depressive symptoms in both adolescents and parents. Evidence-based strategies such as family counseling, communication training, parenting skills programs, and school-based initiatives can effectively mitigate family conflict [48, 66–68]. Family counseling, a proven intervention, offers a secure environment for expressing emotions, enhancing communication, and resolving disputes. In the Chinese context, it can be adapted to emphasize family harmony and cultural values like filial piety, fostering mutual understanding, and a supportive family climate [48, 69]. In addition, effective communication is pivotal in reducing family conflict. Programs teaching communication skills can clarify needs and concerns and improve listening among family members. In Chinese families, where communication may be indirect, training can bridge traditional and modern communication styles, incorporating workshops on active listening, assertive expression, and conflict resolution [42, 48, 70, 71]. Furthermore, parenting programs focusing on positive practices can significantly reduce family conflict by teaching clear expectation-setting, consistent discipline, and emotional support. In China, these programs should integrate cultural norms with modern techniques that promote open dialogue and respect [71]. Moreover, schools can also play a crucial role in preventing family conflict and adolescent depression. School-based programs can include family involvement sessions and provide resources to help families manage conflict more effectively [72, 73].

Although the CFPS provides a large, nationally representative dataset, it may not capture the unique experiences of specific subgroups or communities, limiting the generalizability of the findings. Future research should focus on specific or marginalized groups to better understand the nuances of family conflict and depressive symptoms. The depressive symptoms were measured by the CESD scale with relatively low mean values; therefore, most of the participants were without significant depressive symptoms. In the future, in order to explore the association between depression and family conflict, we should invite psychiatrists using structured questionnaires and semi-structured questionnaires such as DSM-5 or ICD-10 to diagnose depression. Although there are significant differences of depressive symptoms and family conflict across three waves of data, the actual impact of these differences may not be substantial due to the small effect size of one-way ANOVA. We should be cautious in interpreting these results and consider the study design and sample size. Future studies should aim to incorporate a broader range of variables and larger sample sizes to provide a more comprehensive understanding of the factors influencing depressive symptoms and family conflict. Additionally, while the four-year longitudinal data provides valuable insights, longer-term studies are needed to capture more comprehensive trends in mental health. The CLPM used in this study did not account for all possible confounding variables, such as personality traits and external social relationships, which future studies should consider [74, 75]. Moreover, the study did not account for significant external factors, such as the COVID-19 pandemic, which may have affected family dynamics and mental health. Including such factors in future research could provide a more holistic understanding of the relationship between family conflict and depression. In addition, while we evaluated the robustness of our results using three missing value imputation methods, potential biases in the predicted values may arise if the regression model used to fit the real data is misspecified. The selection of the distance metric for matching can introduce bias. Should the distance metric fail to sufficiently capture the similarity between the predicted and observed values, the imputed values may not accurately represent the true missing values. Furthermore, the survey variables were self-reported, which may introduce bias. Parents may underreport depressive symptoms due to stigma, denial, or a desire to present a positive image, leading to an underestimation of the true prevalence and impact on family dynamics. Adolescents, being more emotionally sensitive, may perceive and report family conflict differently from adults, influenced by their emotional state, developmental stage, and temperament. For instance, they might interpret minor disagreements as significant conflicts or report distressing conflicts that parents do not view as serious, potentially inflating the reported frequency of family conflict. Thus, while adolescents’ reports offer a unique perspective on the family’s emotional climate, they should be interpreted cautiously due to potential biases. It is essential to use multiple data sources, such as reports from both adolescents and parents, to provide a more comprehensive and balanced view of family conflict. Conducting validation studies to compare adolescent and parent reports can help identify and correct reporting biases, enhancing the reliability and validity of the findings.

In conclusion, this study substantially advances our understanding of the intergenerational impact of family conflict on depressive symptoms. The findings underscore the pivotal role of family dynamics in mental health, especially in the development of adolescent depressive symptoms. Effective interventions must address both individual symptoms and the broader familial context. Future research should investigate the complex interplay between family conflict, depressive symptoms, and external social influences to develop more comprehensive intervention strategies.

## Electronic supplementary material

Below is the link to the electronic supplementary material.

Supplementary Material 1.

## Data Availability

Data used in the preparation of this article were obtained from the 2020 database of the China Family Panel Studies (CFPS) (https://www.isss.pku.edu.cn/cfps/en/) census, and are held in the Institute of Social Science Survey at Peking University.
