# Supplementary material for: Longitudinal associations between family conflict, intergenerational transmission, and adolescents’ depressive symptoms: evidence from China Family Panel studies (2016–2020)
Source: Child Adolesc Psychiatry Ment Health. 2025 Feb 17;19:10. doi: 10.1186/s13034-025-00866-9 (PMC11834216; doi:10.1186/s13034-025-00866-9)
Supplement: Supplementary file 1 — Supplementary Material 1. [file 13034_2025_866_MOESM1_ESM.docx]

1. **Appendix A1. Process of missing data.**
2. **Table S1 - The imputation of missing data in different ways.**
3. **Table S2 - The sensitivity analysis of the imputation of missing data.**
4. **Figure A1 - Latent Growth Model of CESD scores and family conflict among mothers.**
5. **Figure A2 - Latent Growth Model of CESD scores and family conflict among fathers.**

**Appendix A1. Process of missing data.**

Figure 1 illustrates the data processing flow for the three phases (Wave I, II, and III) of the study. Wave I (2016) initially included 8,427 households. This missing data was considered to be missing completely at random. A Predictive Mean Matching (PMM) method was employed, using matching variables like age, gender, and parents’ incomes to impute missing data, ultimately resulting in a complete dataset for all 2,583 families.

From Wave I (2016) to Wave II (2018), data were missing for 2,040 families and were populated using the same imputation strategy as in Wave I, ensuring that all 2,040 households had complete data by the end of Wave II.

Between Wave II (2018) and Wave III (2020), the same imputation method was applied, resulting in complete data for all 1,922 households by the end of Wave III.

**Table S1 The imputation of missing data in different ways**

| **Variable** | **Imputation (Mean (SD))** | | |
| --- | --- | --- | --- |
|  | **PMM** | **KNN** | **Random Forest** |
| *Adolescent_parent_Quarrels_2016 | 1.16 (2.91) | 1.14 (2.83) | 1.14 (2.82) |
| *Heart_to_heart_talks_2016 | 2.15 (4.49) | 2.14 (4.49) | 2.17 (4.48) |
| *Parental_Quarrels_2016 | 0.79 (2.07) | 0.75 (2.04) | 0.78 (2.03) |
| cesd_t1_2016 | 1.56 (0.70) | 1.59 (0.72) | 1.56 (0.69) |
| cesd_t2_2016 | 1.49 (0.66) | 1.51 (0.68) | 1.49 (0.66) |
| cesd_t3_2016 | 1.38 (0.70) | 1.38 (0.71) | 1.38 (0.70) |
| cesd_t4_2016 | 3.13 (0.83) | 3.14 (0.83) | 3.13 (0.82) |
| cesd_t5_2016 | 1.28 (0.58) | 1.28 (0.58) | 1.28 (0.58) |
| cesd_t6_2016 | 3.26 (0.79) | 3.26 (0.79) | 3.26 (0.78) |
| cesd_t7_2016 | 1.42 (0.60) | 1.43 (0.60) | 1.42 (0.59) |
| cesd_t8_2016 | 1.07 (0.32) | 1.07 (0.32) | 1.07 (0.32) |
| cesd_f_t1_2016 | 1.59 (0.71) | 1.67 (0.75) | 1.59 (0.65) |
| cesd_f_t2_2016 | 1.62 (0.79) | 1.79 (0.95) | 1.64 (0.73) |
| cesd_f_t3_2016 | 1.61 (0.81) | 1.81 (1.00) | 1.62 (0.75) |
| cesd_f_t4_2016 | 2.94 (0.94) | 2.91 (0.98) | 2.93 (0.88) |
| cesd_f_t5_2016 | 1.36 (0.65) | 1.56 (0.90) | 1.38 (0.60) |
| cesd_f_t6_2016 | 3.07 (0.93) | 3.01 (0.99) | 3.06 (0.86) |
| cesd_f_t7_2016 | 1.40 (0.61) | 1.59 (0.85) | 1.43 (0.57) |
| cesd_f_t8_2016 | 1.16 (0.47) | 1.32 (0.77) | 1.16 (0.45) |
| cesd_m_t1_2016 | 1.74 (0.76) | 1.81 (0.83) | 1.74 (0.71) |
| cesd_m_t2_2016 | 1.70 (0.80) | 1.79 (0.91) | 1.69 (0.74) |
| cesd_m_t3_2016 | 1.73 (0.85) | 1.70 (0.85) | 1.73 (0.78) |
| cesd_m_t4_2016 | 2.87 (0.96) | 2.96 (0.97) | 2.88 (0.89) |
| cesd_m_t5_2016 | 1.40 (0.67) | 1.46 (0.76) | 1.43 (0.64) |
| cesd_m_t6_2016 | 3.02 (0.93) | 3.02 (0.94) | 3.01 (0.88) |
| cesd_m_t7_2016 | 1.57 (0.70) | 1.66 (0.79) | 1.58 (0.65) |
| cesd_m_t8_2016 | 1.21 (0.55) | 1.27 (0.65) | 1.20 (0.50) |
| *Adolescent_parent_Quarrels_2018 | 1.08 (2.30) | 1.13 (2.39) | 1.17 (1.92) |
| *Heart_to_heart_talks_2018 | 2.19 (4.66) | 1.75 (3.49) | 1.97 (3.26) |
| *Parental_Quarrels_2018 | 0.79 (2.17) | 0.65 (1.86) | 0.73 (1.72) |
| cesd_t1_2018 | 1.55 (0.66) | 1.80 (0.93) | 1.55 (0.61) |
| cesd_t2_2018 | 1.55 (0.69) | 1.80 (0.94) | 1.56 (0.63) |
| cesd_t3_2018 | 1.49 (0.75) | 1.65 (0.84) | 1.48 (0.68) |
| cesd_t4_2018 | 3.06 (0.82) | 3.11 (0.80) | 3.07 (0.74) |
| cesd_t5_2018 | 1.34 (0.60) | 1.55 (0.83) | 1.34 (0.54) |
| cesd_t6_2018 | 3.20 (0.78) | 3.24 (0.77) | 3.23 (0.70) |
| cesd_t7_2018 | 1.43 (0.60) | 1.62 (0.78) | 1.43 (0.56) |
| cesd_t8_2018 | 1.09 (0.37) | 1.26 (0.70) | 1.08 (0.33) |
| cesd_f_t1_2018 | 1.68 (0.73) | 1.96 (0.97) | 1.69 (0.63) |
| cesd_f_t2_2018 | 1.71 (0.82) | 2.01 (1.04) | 1.72 (0.71) |
| cesd_f_t3_2018 | 1.69 (0.85) | 1.93 (1.01) | 1.65 (0.73) |
| cesd_f_t4_2018 | 2.84 (0.94) | 2.93 (0.95) | 2.86 (0.82) |
| cesd_f_t5_2018 | 1.47 (0.74) | 1.75 (1.01) | 1.43 (0.63) |
| cesd_f_t6_2018 | 2.98 (0.90) | 3.02 (0.92) | 3.02 (0.79) |
| cesd_f_t7_2018 | 1.49 (0.66) | 1.73 (0.91) | 1.46 (0.56) |
| cesd_f_t8_2018 | 1.20 (0.53) | 1.38 (0.77) | 1.17 (0.45) |
| cesd_m_t1_2018 | 1.84 (0.78) | 2.04 (0.92) | 1.85 (0.71) |
| cesd_m_t2_2018 | 1.80 (0.85) | 2.03 (0.99) | 1.81 (0.77) |
| cesd_m_t3_2018 | 1.84 (0.91) | 2.06 (1.03) | 1.82 (0.82) |
| cesd_m_t4_2018 | 2.76 (0.92) | 2.78 (0.95) | 2.79 (0.84) |
| cesd_m_t5_2018 | 1.50 (0.74) | 1.71 (0.93) | 1.50 (0.67) |
| cesd_m_t6_2018 | 2.94 (0.90) | 2.97 (0.91) | 2.98 (0.81) |
| cesd_m_t7_2018 | 1.63 (0.73) | 1.87 (0.92) | 1.65 (0.66) |
| cesd_m_t8_2018 | 1.26 (0.61) | 1.48 (0.86) | 1.24 (0.54) |
| *Adolescent_parent_Quarrels_2020 | 1.84 (3.84) | 1.15 (3.15) | 1.50 (1.74) |
| *Heart_to_heart_talks_2020 | 2.08 (3.62) | 1.67 (2.60) | 2.34 (2.06) |
| *Parental_Quarrels_2020 | 1.39 (3.81) | 0.71 (2.16) | 0.93 (1.58) |
| cesd_t1_2020 | 1.69 (0.74) | 1.95 (0.85) | 1.72 (0.62) |
| cesd_t2_2020 | 1.59 (0.74) | 1.91 (0.90) | 1.58 (0.63) |
| cesd_t3_2020 | 1.55 (0.80) | 1.94 (1.10) | 1.59 (0.67) |
| cesd_t4_2020 | 3.10 (0.84) | 3.23 (0.80) | 3.09 (0.71) |
| cesd_t5_2020 | 1.47 (0.73) | 1.85 (1.04) | 1.50 (0.61) |
| cesd_t6_2020 | 3.20 (0.80) | 3.16 (0.74) | 3.16 (0.66) |
| cesd_t7_2020 | 1.56 (0.68) | 1.83 (0.84) | 1.56 (0.58) |
| cesd_t8_2020 | 1.15 (0.46) | 1.41 (0.78) | 1.15 (0.39) |
| cesd_f_t1_2020 | 1.70 (0.77) | 2.23 (1.13) | 1.77 (0.65) |
| cesd_f_t2_2020 | 1.71 (0.85) | 2.30 (1.19) | 1.78 (0.71) |
| cesd_f_t3_2020 | 1.70 (0.87) | 2.17 (1.17) | 1.73 (0.73) |
| cesd_f_t4_2020 | 2.90 (0.97) | 3.14 (0.92) | 2.93 (0.80) |
| cesd_f_t5_2020 | 1.50 (0.76) | 1.94 (1.06) | 1.49 (0.62) |
| cesd_f_t6_2020 | 3.01 (0.96) | 3.19 (0.91) | 3.02 (0.79) |
| cesd_f_t7_2020 | 1.52 (0.71) | 1.98 (1.03) | 1.56 (0.59) |
| cesd_f_t8_2020 | 1.24 (0.61) | 1.64 (1.04) | 1.23 (0.50) |
| cesd_m_t1_2020 | 1.85 (0.80) | 2.04 (1.04) | 1.77 (0.70) |
| cesd_m_t2_2020 | 1.80 (0.86) | 2.24 (1.12) | 1.80 (0.73) |
| cesd_m_t3_2020 | 1.83 (0.91) | 2.07 (1.07) | 1.85 (0.77) |
| cesd_m_t4_2020 | 2.83 (0.96) | 2.95 (0.94) | 2.81 (0.82) |
| cesd_m_t5_2020 | 1.50 (0.73) | 1.88 (1.06) | 1.47 (0.61) |
| cesd_m_t6_2020 | 2.95 (0.94) | 2.94 (0.97) | 2.89 (0.79) |
| cesd_m_t7_2020 | 1.63 (0.70) | 1.97 (1.00) | 1.58 (0.58) |
| cesd_m_t8_2020 | 1.30 (0.63) | 1.64 (1.01) | 1.28 (0.53) |

*Notes*. * refers to the untransformed original frequency; All CESD-8 questions showed above retain their original scores (ranging from 1 to 4) as recorded in the CFPS database (However, in the manuscript, CESD-A, CESD-F, and CESD-M scores have been transformed into a scale ranging from 0 to 24)..

**Table S2 The sensitivity analysis of the imputation of missing data.**

| Model | Variables/Parameter | Predictor | PMM | | | | KNN | | | | RF | | | |
| --- | --- | --- | --- | --- | --- | --- | --- | --- | --- | --- | --- | --- | --- | --- |
|  |  |  | Estimate | Std.Err | z-value | P(>\|z\|) | Estimate | Std.Err | z-value | P(>\|z\|) | Estimate | Std.Err | z-value | P(>\|z\|) |
| CLPM | cesd_child_2018 | cesd_chld_2016 | 0.18 | 0.027 | 6.796 | <0.001 | 0.18 | 0.027 | 6.796 | <0.001 | 0.187 | 0.028 | 6.752 | 0 |
|  |  | Conflict_2016 | 0.164 | 0.056 | 2.933 | 0.003 | 0.164 | 0.056 | 2.933 | 0.003 | 0.17 | 0.058 | 2.946 | 0.003 |
|  |  | cesd_fthr_2016 | 0.035 | 0.021 | 1.625 | 0.104 | 0.035 | 0.021 | 1.625 | 0.104 | 0.002 | 0.022 | 0.069 | 0.945 |
|  |  | cesd_mthr_2016 | 0.033 | 0.02 | 1.634 | 0.102 | 0.033 | 0.02 | 1.634 | 0.102 | 0.033 | 0.021 | 1.569 | 0.117 |
|  |  |  |  |  |  |  |  |  |  |  |  |  |  |  |
|  | cesd_child_2020 | cesd_chld_2018 | 0.2 | 0.028 | 7.171 | <0.001 | 0.2 | 0.028 | 7.171 | <0.001 | 0.156 | 0.028 | 5.63 | <0.001 |
|  |  | Conflict_2018 | 0.055 | 0.064 | 0.864 | 0.387 | 0.055 | 0.064 | 0.864 | 0.387 | 0.109 | 0.073 | 1.495 | 0.135 |
|  |  | cesd_fthr_2018 | 0.044 | 0.023 | 1.881 | 0.06 | 0.044 | 0.023 | 1.881 | 0.06 | 0.07 | 0.023 | 3.01 | 0.003 |
|  |  | cesd_mthr_2018 | 0.043 | 0.022 | 2.001 | 0.045 | 0.043 | 0.022 | 2.001 | 0.045 | 0.076 | 0.021 | 3.608 | <0.001 |
|  |  |  |  |  |  |  |  |  |  |  |  |  |  |  |
|  | Conflict_2018 | cesd_chld_2016 | 0.03 | 0.012 | 2.566 | 0.01 | 0.03 | 0.012 | 2.566 | 0.01 | 0.014 | 0.011 | 1.304 | 0.192 |
|  |  | cesd_fthr_2016 | 0.01 | 0.009 | 1.055 | 0.291 | 0.01 | 0.009 | 1.055 | 0.291 | 0.005 | 0.008 | 0.64 | 0.522 |
|  |  | cesd_mthr_2016 | -0.002 | 0.009 | -0.232 | 0.816 | -0.002 | 0.009 | -0.232 | 0.816 | 0.004 | 0.008 | 0.493 | 0.622 |
|  |  | Conflict_2016 | 0.125 | 0.025 | 5.024 | <0.001 | 0.125 | 0.025 | 5.024 | <0.001 | 0.165 | 0.022 | 7.523 | <0.001 |
|  |  |  |  |  |  |  |  |  |  |  |  |  |  |  |
|  | Conflict_2020 | cesd_chld_2018 | 0.032 | 0.011 | 2.861 | 0.004 | 0.032 | 0.011 | 2.861 | 0.004 | 0.012 | 0.007 | 1.653 | 0.098 |
|  |  | cesd_fthr_2018 | 0 | 0.009 | -0.052 | 0.958 | 0 | 0.009 | -0.052 | 0.958 | -0.001 | 0.006 | -0.179 | 0.858 |
|  |  | cesd_mthr_2018 | 0.009 | 0.009 | 1.058 | 0.29 | 0.009 | 0.009 | 1.058 | 0.29 | 0.002 | 0.005 | 0.332 | 0.74 |
|  |  | Conflict_2018 | 0.07 | 0.025 | 2.768 | 0.006 | 0.07 | 0.025 | 2.768 | 0.006 | 0.085 | 0.019 | 4.481 | <0.001 |
|  |  |  |  |  |  |  |  |  |  |  |  |  |  |  |
| LGM—-A | i1 | | 4.367 | 0.172 |  | <0.001 | 4.846 | 0.233 |  | <0.001 | 4.427 | 0.247 |  | <0.001 |
|  | s1 | | 0.532 | 0.139 |  | <0.001 | 1.368 | 0.224 |  | <0.001 | -0.248 | 0.193 |  | 0.199 |
|  | i2 | | 2.164 | 0.079 |  | <0.001 | 2.191 | 0.102 |  | <0.001 | 2.329 | 0.113 |  | <0.001 |
|  | s2 | | 0.196 | 0.06 |  | 0.001 | 0.062 | 0.074 |  | 0.404 | 0.667 | 0.071 |  | <0.001 |

**Figure A1 - Latent Growth Model of mothers’ depressive symptoms and family conflict among mothers.**

**
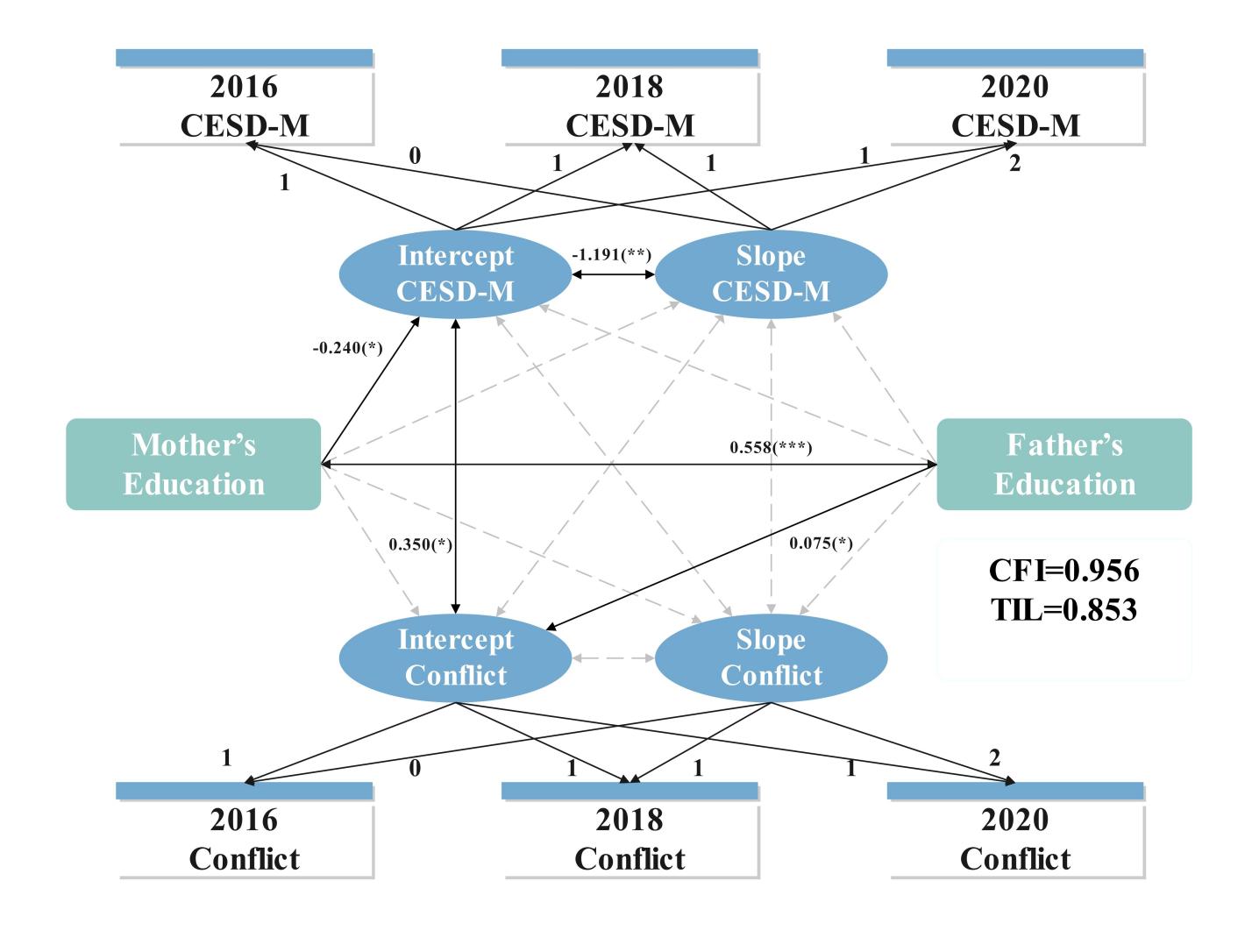
**

*Notes.* CESD-M, mothers’ depressive symptoms; CFI, comparative fit index; TIL, tucker-lewis index.

**Figure A2 - Latent Growth Model of fathers’ depressive symptoms and family conflict among fathers.**

**
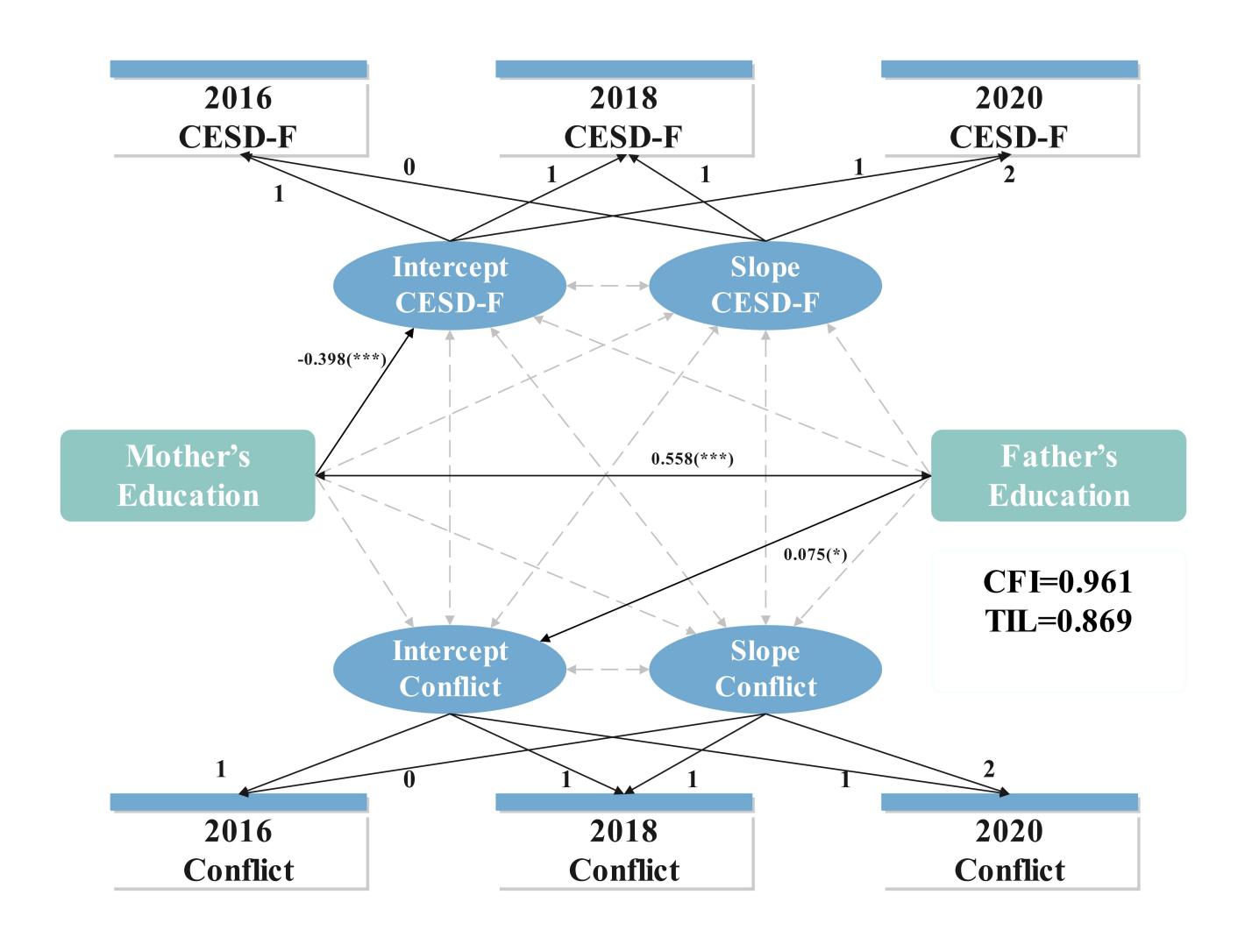
**

*Notes.* CESD-F, fathers’ depressive symptoms; CFI, comparative fit index; TIL, tucker-lewis index.
